# Telephone - delivered quality of life after 365 male stress urinary incontinence (SUI) operations

**DOI:** 10.1590/S1677-5538.IBJU.2015.0194

**Published:** 2016

**Authors:** Katharina Maria Bretterbauer, Erik Randall Huber, Mesut Remzi, Wilhelm Huebner

**Affiliations:** 1 Landesklinikum Mistelbach – Urology Mistelbach, Lower Austria, Austria; 2 Landesklinikum Korneuburg – Urology Korneuburg, Austria

**Keywords:** Urinary Incontinence, Prostate, Neoplasms

## Abstract

**Objectives:**

To assess patient satisfaction and quality of life and factors that may be related to these outcomes.

**Materials and Methods:**

Between 2000 and 2008 a retrospective chart review and telephone survey of all surgeries for male SUI was performed. Average age at times of operation was 69.4 ± 7.4 (median 69). As part of the survey 270 of 365 patients were available (response rate: 74%). The average follow up time (from operation to telephone survey) was 34.8 ± 22.8 months (median 32).

**Results:**

Pad use per day improved significantly after operation from 6.23±5.3 to 1.61±2.92 pads/day (p=0.001). 74.7% (n=198) declared to be continent with one safety pad and 87.7% (n=236) confirmed the postoperative improvement of incontinence. 189 (70.5%) patients were “very satisfied” and “satisfied”. In 81% (n=218) the expectation in operation could be met, therefore 84.3% (n=226) would undergo it again and 90.3% (n=243) would recommend it to others. Lower age (rs=0.211), few postoperative pads per day (rs=0.58), high reduction of pads (rs=-0.35) and physical activity level (rs=0.2) correlate significantly with better satisfaction.

**Conclusions:**

Eighty-seven pint seven percent (87.7%) of our incontinence operations (n=236) lead to an improvement, which is independent from the number of prior incontinence operations and preoperative pad count. The postoperative quality of life remains constant over the observed follow up time. Certain subgroups of patients (younger age, high physical activity level, large reduction of pads) demonstrated superior satisfaction rates.

## INTRODUCTION

Stress urinary incontinence (SUI) is a common adverse event of prostate surgery associated with significant alteration in quality of life for the patients and is a frustrating problem for the urologist (1). The incidence of post-prostatectomy incontinence (PPI) reported in the literature ranges between 5.0% to 48% (2). PPI continues to be a bothersome complication after surgery although the incidence has decreased with the better preserving of neurovascular bundles and improved operative techniques (3). SUI can also occur in 1% of patients after laparoscopic or open surgery for prostate hyperplasia (4). It is reported that approximately 10% of the affected men will consider their SUI bothersome enough to seek medical attention (5). The impact of SUI on quality of life should be observed on long term (>5 years) as Resnick et al. underlined (6). Initial management is usually conservative and includes the use of pads, pelvic floor exercise, penile clamps or collecting systems. In patients with persistent post-prostatectomy SUI surgical treatment is recommended. In general, a surgery should be recommended if conservative treatments fail after 6-12 months (2). The surgical armamentarium for SUI in men ranges from bulking agents, Pro adjustable continence therapy (ACT), various sling insertions to artificial urinary sphincters (AUS). To our knowledge there is no publication that compared the objective and subjective outcome of different procedures performed in one department when evaluated by an outsider of the clinic and thus preventing a major bias -namely the patient who sometimes tries to please the surgeon by giving a more positive feedback than the physical situation would allow (7).

## MATERIALS AND METHODS

A retrospective chart review of all patients undergoing surgeries for male urinary incontinence between February 2000 and October 2008 was performed. Overall 365 patients that had undergone surgeries for male urinary incontinence were identified and included into our review. Patient's data for preoperative daily pad use, age at time of operation, duration of operation, time from onset of incontinence to definite treatment and time of last follow-up in our department were collected. A telephone questionnaire was conducted to assess patient's follow-up data.

Being a referral center for male SUI this cohort includes 116 patients (43%) with proceeding failed treatment attempts, thus representing a difficult patient's selection. All identified patients were included into a telephone survey performed by an external contractor, who had access to the collected patient's data.

In those patients in which the number was absent or changed we used an online telephone book or called for phone assistance. The resident's registration office was used to identify patients that where still not reachable and might have been deceased in the meanwhile.

Data to all answers were documented on Excel 2003 (Microsoft®). The statistic was performed on SPSS 20. Parametric tests (t test, paired t Test, ANOVA) were used for normally distributed data. Non-parametric tests (Wilcoxon rank sum test, Kruskal-Wallis test) were used for data with slate distribution and chi-square test for categorical data. Bivariate correlation analysis was performed with spearman's rank correlation.

## RESULTS

The main cause for male urinary incontinence in this study was radical prostatectomy in 89.3% (n=326), TURP in 7% (n=26) and other operations in 3.7% (n=13).

The basic data of 365 patients that had undergone surgeries for male urinary incontinence are shown in Table-1. Two hundred eighty-five (78.3%) patients could be reached by phone. Of these 15 (4.4%) declined participation in a telephone survey concerning their state of continence. Thirty-six (9.9%) patients were deceased at the time of the survey and 43 (11.8%) patients could not be reached by any means, which in part might be due to the fact that we get referrals from foreign countries as well. In 270 (74%) patients the survey was conducted.

**Table 1:** Shows basic data of 365 patients.

	Mean	St.deviation	Median	Range
Preoperative daily pad use per day	6.3	5.1	4.5	0-20
Age at time of SUI operation (years)	69.4	7.4	69	31-89
Duration of SUI operation (minutes)	41.6	31.6	30	9-230
Length of hospitalisation (days)	6.7	3.4	6	2-33
Time from onset of incontinence to definite treatment (years)	4.2	3.3	3.3	0.1-22.4
Time from SUI operation to telephone survey (months)	34.8	22.8	32	0.24-88

The performed incontinence surgeries were composed of 149 (55.3%) cases of Pro ACT, 57 (21.1%) cases of Argus slings, 54 (20.0%) cases of AUS, 10 (3.7%) cases of Flow Secure, Invance or Reemex. 78.1% of operations were performed by the same surgeon.

The results of the telephone survey questionnaire are shown in Table-2.

**Table 2:** Questions and results of the telephone survey.

		n (%)	Mean (SD)	Median (IQR)
**1a. What is your daily pad use today including a safety pad for activities?**		267 (100)	1.6 (2.9)	1 (2)
(Pads per day)				
**1b. How would you define your continence?**		265 (100)	2 (1.2)	2 (2)
	1 - Excellent, Dry	123 (46.4)		
	2 - Good	75 (28.3)		
	3 - Satisfactory	37 (14.0)		
	4 - Poor	15 (5.7)		
	5 - Unsatisfactory, Complete Incontinent	15 (5.7)		
**1c. How is your state of incontinence compared to before operation?**		269 (100)		
	Better	236 (87.7)		
	Same	24 (8.9)		
	Worse	9 (3.3)		
**2. What is your overall satisfaction with the procedure ?**		268 (100)	2.0 (1.3)	2 (2)
	1 - Excellent	129 (48.1)		
	2 - Good	60 (22.4)		
	3 - Satisfactory	43 (16)		
	4 - Poor	13 (4.9)		
	5 - Unsatisfactory	23 (8.6)		
**3a. Did the incontinence surgery meet your expectations?**		269 (100)		
	Yes	218 (81)		
	No	51 (19)		
**3b. Would you undergo the same operation again?**		268 (100)		
	Yes	226 (84.3)		
	No	42 (15.7)		
**3c. Would you recommend the operation to others?**		269 (100)		
	Yes	243 (90.3)		
	No	26 (9.7)		
**4. How physically active are you?**		267 (100)	2 (1.1)	2 (2)
	1 - High Physical Activitiy Level	121 (45.3)		
	2 - Very Active	72 (27)		
	3 - Active	39 (14.6)		
	4 - Little Active	24 (9.0)		
	5 - Low Physical Activity Level/Immobile	11 (4.1)		

We compared the preoperative pad count per day 6.23±5.3 with postoperative 1.61±2.92 and analyzed the change of pad use/day. A statistically significant difference in pad usage could be found (p=0.001). Two hundred thirty-five men (88.7%) assessed their postoperative incontinence situation with “satisfactory” or better.

Furthermore, we divided the patients into 4 grades of incontinence (dry (0 pad), mild (1-2 pads/day), moderate (3-5 pads/day) and severe (>5 pads/day)) and tracked the changes in satisfaction rate and reduction of pads within these groups.

Before the operation zero patients were dry (0 pad), 64 (25.6%) suffered from mild incontinence (1-2 pads/day), 100 (40%) moderate (3-5 pads/ day) and 86 (34.4%) severe (>5 pads/day). After the operation 75 (27.9%) patients were dry (0 pad), 138 (51.3%) had mild incontinence (1-2 pads/day), 45 (16.7%) moderate (3-5 pads/day) and 11 (4.1%) severe (>5 pads/day).

The best results in satisfaction rate were reached in patients who were dry or had mild incontinence. A significant difference in satisfaction depending on the 4 grades of incontinence can be observed (p=0.001): Patients with severe preoperative incontinence have the highest reduction in pad use per day on average 8 pads/day. Two hundred thirty-two patients (86.5%) rated their satisfaction rate with “satisfactory” or better.

In Table-3 we looked at different methods of incontinence operations and it can be seen, that there is a significant difference in preoperative pads per day (p=0.001), reduction of pads per day (p=0.008) as well as satisfaction rate (p=0.04) in favor of the AUS subgroup.

**Table 3:** Illustrates an overview over the collected data accordin to different methods of SUI operations.

	Pro ACT	Argus sling	AUS	Significance
n=270	149	57	54	
preoperative pads/day	5.3±4.6	6.0±5.0	8.8±6.8	0.001
postoperative	1.4±2.6	1.9±4.0	1.7±2.9	0.85
pads/day				
reduction of pads/day	3.8±4.7	4.1±6.4	7.1±7.5	0.008
satisfaction rate	2.1±1.3	2.0±1.3	1.7±1.0	0.04
met expectation	119 (79.9%)	43 (76.8%)	49 (90.7%)	0.18
surgery again	125 (84.5%)	42 (75.0%)	52 (94.4%)	0.05
recommend it again	137 (91.1%)	47 (83.9%)	50 (92.6%)	0.34

A high reduction of pads correlates with a good satisfaction rate (rs=-0.35) and therefore people with AUS are more satisfied (p=0.04) and would recommend it again (p=0.04) compared to patients with other treatment operations.

Moreover, it must be stated that 71.1% of our patients with Pro ACT need 0-1 pads/day postoperatively and 76% of Pro ACTs were performed by the same surgeon.

Forty-three percent of our patients had one or more prior incontinence operations. To identify any differences in outcome as a function of prior operations for SUI we classified the survey patients into 3 groups: no prior SUI operation, one prior and more than one. Results are shown in Table-4.

**Table 4:** Summary of collected data according to the number of prior SUI operations.

Number of prior SUI operations	No prior	1 prior	>1 prior	Significance
n= 270	154	70	46	
Better Continence	136 (88.3%)	61 (87%)	40 (87%)	ns
Pads/Day	1.4 ± 2.6	1.8 ± 3.5	2 ± 3.1	ns
Reduction Of Pads/Day	5.1 ± 5	5.7 ± 5.5	6.4 ± 4.8	ns
Satisfaction Rate	2 ± 1.3	2 ± 1.2	2.1 ± 1.4	ns
Met Expectations	128 (83.1%)	53 (76.8%)	37 (80.4%)	ns
Surgery Again	133 (86.4%)	54 (78.3%)	39 (86.7%)	ns
Recommend It Again	143 (92.9%)	61 (88.4%)	39 (84.8%)	ns

To analyze the differences in the survey results for different times of follow-up we divided into 3 follow-up groups: a short term follow-up group (<12 months), an intermediate follow-up of (12-36 months) and a long term follow-up group of (>36 months). Table-5 presents the comparison for the different parameters of the survey.

**Table 5:** Collection of data dependent on follow up time.

	Short follow-up (<12 Months)	Intermediate follow Up (12- 36 months)	Long Term Follow-up (>36 months)	Significance
n=	63	95	112	
Better continence	57 (90.5%)	82 (86.3%)	97 (86.6%)	ns
Pads/day	1.2 ± 1.3	1.7 ± 3.2	1.8 ± 3.4	ns
Satisfaction rate	2 ± 1.24	2.1 ± 1.3	2 ± 1.2	ns
Met expectations	50 (79.4%)	76 (80.9%)	92 (82.1%)	ns
Surgery again	53 (84.1%)	81(86.2%)	92 (82.9%)	ns
Recommend it again	55 (87.3%)	86 (91.5%)	102 (91.9%)	ns

As the physical activity level plays a major role in PPI, patients were also asked about their physical activity. One hundred twenty-one (45.3%) considered themselves as “highly physically active”; 72 (27%) as “very active”; 39 (14.4%) as “active”; 24 (9%) as “little active” and 11 (4.1%) as “less physically active/immobile”.

In order to see if activity had an impact on surgery outcome we separated all patients into two different activity groups and compared satisfaction rate and pad use at time of the survey as well as the change in pad use (Table-6).

**Table 6:** This chart shows the collected data depending on physical activity level.

	High physical activity level	Low physical activity level/ immobile	Significance
n=	218	29	
age (years)	68 ± 7.1	70.3 ± 7.9	ns
better continence	177 (91.7%)	57 (77%)	0.005
preoperative pads/day	6.1 ± 5.3	6.3 ± 5	ns
postoperative pads/day	1.2 ± 2.3	2.6 ± 4	0.007
reduction of pads/ day	4.9 ± 5.5	3.7 ± 6.6	ns
satisfaction rate	1.9 ± 1.2	2.8 ± 1.4	0.026
met expectation	165 (85.5%)	52 (70.3%)	0.004
surgery again	169 (87.6%)	56 (76.7%)	0.029
recommend it again	180 (93.3%)	62 (83.8%)	0.017

We searched for variables which correlate with a better satisfaction rate and found out that lower age (rs=0.211), few postoperative pads per day (rs=0.58), a high reduction of pads per day (rs=-0.35) and high physical activity level correlate significantly with a better satisfaction rate. Factors for low postoperative pads per day are low age at time of SUI operation (rs=0.17), few incontinence operations (rs=0.16) and few pads before the operation (rs=0.18). The connection between these is weak. A large number of incontinence operations (rs=0.18) or preoperative pads per day (rs=0.16) have a positive correlation with the length of hospitalization. The present correlations are significant at the 0.01 level (2-tailed).

## DISCUSSION

Male incontinence has gained significant interest in the past 10 years and has become an essential part of most major urology conferences. The optimal postoperative results depend on many individual patient, device and surgeon-related factors. Comiter et al. claimed that it is not clear which device should be offered to which patient, because no single device should be exclusively considered the gold-standard option for PPI therapy (8).

As Martin et al. described, comparison of different methods in different set-ups and for different initial situations continues to be difficult, as we lack standardized methods and validated questionnaires used widely to evaluate the incontinence status in a particular patient (9, 10).

In addition, our patients may have different habits and expectations. In a study by Kumar et al. when men with PPI are offered the choice of a mechanical device (AUS) vs. a male sling, they are willing to go against the surgeon's recommendation to avoid a mechanical device (11).

Furthermore, different systems are difficult to compare, as indications as well as contraindications depend on various factors. Therefore, it is not easy to predict the subjective outcome after surgery for male incontinence in a particular patient (12).

According to the EAU guidelines the AUS is the therapy of choice for moderate to severe incontinence (13). Studies have shown that the success rates of AUS are highest compared with all other treatment options for male SUI (2). It must be indicated, that AUS implantation carries a well known risk of revision surgery secondary to infection, erosion, urethral atrophy and mechanical failure (14, 15). However, it must be stated that the comparison of pads/day between operation procedures is misleading because of the fact that patients with severe incontinence would get an AUS. Unsurprisingly, there was a significant difference in preoperative pad count, reduction of pads as well as satisfaction rate in favor of the AUS subgroup.

It was not our intent to compare different surgical techniques but rather get an idea which patients would subjectively profit most from surgery for their incontinence. To compare this, a prospective study design would be needed. Furthermore, the study population is non-homogenous, no validated quality of life questionnaire was used and 63 (23.3%) patients had a follow-up time less than one year. These mentioned points can also be seen as a limitation of this study.

In the context of the survey 84.3% claimed to undergo the operation again, but nearly 90.3% (n=243) would recommend it to others. Most of the people questioned argued with the uniqueness of mankind. Walsh et al. investigated the satisfaction after AUS implantation in irradiated patients, asked the same questions above and found the opposite result. Eighty-nine percent would undergo the surgery again, but only 87% would recommend it to a friend (16).

Men with severe preoperative incontinence have the highest reduction of pads postoperative. Interestingly enough preoperative pad count does not correlate with better postoperative satisfaction rate.

Furthermore, no difference in satisfaction rate can be shown according to the number of prior incontinence operations even though pads/day is slightly higher at time of survey in patients with prior operations for SUI. It must be highlighted that a low number of prior incontinence operations correlates with low postoperative pad count, but not with satisfaction rate. In addition, it is noteworthy that patients with more than one prior operation would undergo surgery again if necessary.

Seventy-one point one percent (71.1%) of our patients with Pro ACT need 0-1 pads/day. These good results compared to literature can be explained by the fact that the success rate increases with experience. In our study, 76% of Pro ACTs were performed by the same surgeon (17).

Our collected data makes it possible to compare postoperative pad count and satisfaction rate over various follow-up times (range: 1-88 months) and it revealed that although the pad count slightly increased, the satisfaction rate stayed constantly.

It is worth mentioning that there was no significant difference in pad use per day prior to surgery between the low and high physical activity level group. Besides, the highly active group had a significant improvement in postoperative pad usage and satisfaction rate. People who regard themselves as highly active, are typically younger and have a better outcome than people with a low activity self awareness. By asking specifically about their desire of activity, we think this is not due to a recall bias and people with a higher postoperative incontinence rate are just more prone to dismiss activities that could result in incontinence. The qualities young and active are probably linked together due to the fact that younger men are more active than older and may also be more confident with their lives. The reason why younger and active patients will profit more remains unclear. It might be due to the fact that a higher muscular tone will also improve pelvic floor function. Furthermore, their grade of injury to the continence mechanism may be overrated compared to the inactive group due to higher demands.

A correlation for low postoperative pad count was found for young age at time of SUI operation, few incontinence operations and few pads before SUI operation. However, the satisfaction rate was only influenced by low age, high reduction in pad usage, physical activity level and few postoperative pads. Hence, the amount of preoperative incontinence operations correlates with low postoperative pads but not with the satisfaction rate.

## CONCLUSIONS

Eighty-seven point seven percent (87.7%) of our incontinence operations (n=236) lead to an improvement, which is irrespective of the number of prior incontinence operations and preoperative pad count. The postoperative satisfaction rate remains constant over the observed follow-up time. Certain subgroups of patients (younger age, high physical activity level, large reduction of pads) demonstrated superior satisfaction rates.
